# SARS-CoV-2 infection and smoking: What is the association? A brief review

**DOI:** 10.1016/j.csbj.2021.03.023

**Published:** 2021-03-23

**Authors:** Ishita Gupta, Muhammad Umar Sohail, Khaled E. Elzawawi, Ahmed H. Amarah, Semir Vranic, Maha Al-Asmakh, Ala-Eddin Al Moustafa

**Affiliations:** a College of Medicine, QU Health, Qatar University, Doha, Qatar; b Biomedical and Pharmaceutical Research Unit, QU Health, Qatar University, Doha, Qatar; c Proteomics Core, Weill Cornell Medicine, Qatar Foundation — Education City, P. O. Box 24144, Doha, Qatar; d Department of Biomedical Sciences, College of Health Sciences, QU Health, Qatar University, Doha, Qatar; e Biomedical Research Center, QU Health, Qatar University, Doha, Qatar

**Keywords:** ACE2, angiotensin-converting enzyme-2, ACEIs, Angiotensin‐converting enzyme inhibitors, ADAM17, ADAM metallopeptidase domain 17, ALCAM, activated leukocyte cell adhesion molecule, Ang, angiotensin, ARBs, angiotensin receptor blockers, ARDS, acute respiratory distress syndrome, BatCoV, bat coronavirus, CLDN7, claudin 7, COPD, chronic obstructive pulmonary disease, COVID-19, coronavirus disease 2019, CTNNB1, catenin beta 1, ERK, extracellular signal-regulated kinases, HDAC6, histone deacetylase 6, HIV-1, human immunodeficiency virus 1, hrsACE2, human recombinant soluble ACE-2, IFN, Interferons, IPF, Idiopathic pulmonary fibrosis, IR, Ionizing radiation, JNK, c-Jun N-terminal kinase, MERS, middle-East respiratory syndrome, MCN, mucin, R0, R-nought, nAChR, α7 nicotinic acetylcholine receptor, NO, nitric oxide, RAS, renin-angiotensin, RR, relative risk, SARS-CoV-2, severe acute respiratory syndrome coronavirus, TJP3, tight junction protein 3, TMPRSS, transmembrane serine protease, SARS-CoV-2, Coronavirus, COVID-19, Smoking, Lung disease, Oral disease

## Abstract

Susceptibility to severe illness from COVID-19 is anticipated to be associated with cigarette smoking as it aggravates the risk of cardiovascular and respiratory illness, including infections. This is particularly important with the advent of a new strain of coronaviruses, the severe acute respiratory syndrome coronavirus (SARS-CoV-2) that has led to the present pandemic, coronavirus disease 2019 (COVID-19). Although, the effects of smoking on COVID-19 are less described and controversial, we presume a link between smoking and COVID-19. Smoking has been shown to enhance the expression of the *angiotensin-converting enzyme-2* (*ACE-2*) and *transmembrane serine protease 2* (*TMPRSS2*) key entry genes utilized by SARS-CoV-2 to infect cells and induce a ‘cytokine storm’, which further increases the severity of COVID-19 clinical course. Nevertheless, the impact of smoking on *ACE-2* and *TMPRSS2* receptors expression remains paradoxical. Thus, further research is necessary to unravel the association between smoking and COVID-19 and to pursue the development of potential novel therapies that are able to constrain the morbidity and mortality provoked by this infectious disease. Herein we present a brief overview of the current knowledge on the correlation between smoking and the expression of SARS-CoV-2 key entry genes, clinical manifestations, and disease progression.

## Highlights

• The link between smoking and the expression of SARS-CoV-2 key entry genes is discussed.
• Smoking-related cardiac and respiratory diseases are risk factors for COVID-19.
• The impact of smoking on *ACE-2* and *TMPRSS2* receptors expression is controversial.

## Graphical abstract

**Figure:**
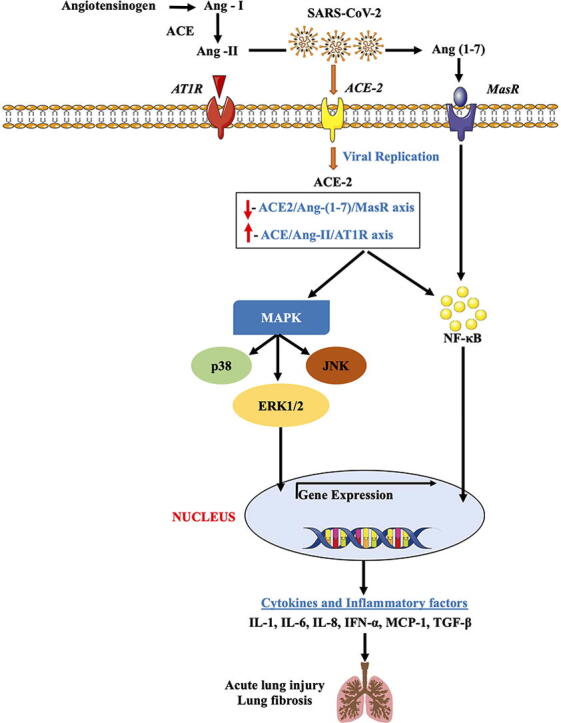

## 1 Introduction

The current outbreak of a novel severe acute respiratory syndrome coronavirus 2 (SARS-CoV-2) causes a severe respiratory illness called coronavirus disease 2019 (COVID-19). SARS-CoV-2 is an enveloped RNA virus with high zoonotic potential. Genetic analysis revealed that SARS-CoV-2 belongs to the *Betacoronavirus* genus and the *Sarbecovirus* subgenus, indicating that it originated from the bat coronavirus (BatCoV RaTG13) [1]. The initial epidemic, which began in Wuhan, China, continued seeding secondary chains through international travel and propagated to over 180 countries and territories [2]. As of October 2020, more than 34 million confirmed cases have been reported, including over one million deaths, with a rising number of cases reported in India, the United States, and Brazil [3]. SARS-CoV-2 is an extremely contagious infection with interpersonal transmission by aerosols and fomites; the virus remains environmentally active in the atmosphere and on surfaces for several days [4]. These features make it possible for the virus to spread rapidly and cause a global pandemic.

The outbreak was initially presented in the form of pneumonia of unknown etiology. As the epidemic evolved into a global pandemic, it became clear that the SARS-CoV-2 infection contributes to multi-organ disease, with a high incidence of acute respiratory distress syndrome (ARDS) and digestive tract complications [5]. SARS-CoV-2 has a broad spectrum of clinical manifestations ranging from asymptomatic to multi-organ dysfunction [6]. The disease is graded into mild, moderate, severe, and critical, depending on the severity of the presentation, while the most common symptoms include fever, cough, diarrhea, and fatigue [6]. Based on the R-nought (R0) values, it is considered that SARS-CoV-2 is more contagious than previous coronavirus pandemics (SARS or MERS) [7], [8]. Certain population segments are at a higher risk of fatality, including the elderly, immunocompromised, comorbid patients, and smokers [9], [10]. In general, smokers are more likely to get respiratory diseases than non-smokers [11]. Smokers also have twice the risk of acquiring influenza infection, tuberculosis, and bacterial pneumonia [12], [13]. A *meta*-analysis revealed that 22% (n = 31/139) of current smokers and 46% (n = 13/28) of ex-smokers had severe complications, including ARDS [14]. However, relative risk (RR) analysis showed that current smokers were 1.45 times more likely than former and never-smoker patients to have severe complications [14], [15], [16]. Nevertheless, the role of smoking in COVID-19 remains controversial [14], [17], [18]. This review will focus on the relationship between smoking and receptors of SARS-CoV-2 and impact on COVID-19 leading to tissue damage and diseases especially in the lungs.

## 2 SARS-CoV-2 and its key entry genes

The Latin word “corona” ascended from the crown-like appearance of the coronavirus structural images. The large type 1 transmembrane spike (S) glycoproteins attribute to the virus's corona shape. The S glycoprotein comprises two distinct functional domains (S1 and S2) that are assumed to mediate host cell binding, membrane fusion, and virus entry [19]. The S1 domain contains receptor-binding sites for *angiotensin-converting enzyme-2* (*ACE2*) and is responsible for virus entry into host cells [20]. The S2 domain enables the fusion of the virus membrane and the host cell, essential for cell infiltration [21].

There is still a limited understanding of the pathogenic pathways of SARS-CoV-2. Viral particles infiltrate the host respiratory epithelial cell through metallopeptidase cell identification. Host cell enzymes must cleave the S glycoprotein at two different sites for coronaviruses to enter target cells, thereby presenting potential drug targets. Several host enzymes are known to activate SARS-CoV-2, including cathepsin L, furin, and transmembrane serine protease 2 (TMPRSS2), TMPRSS4, TMPRSS11A, and TMPRSS11D [22]. However, *TMPRSS2* and *furin* play key roles in the proteolytic activation of SARS-CoV-2 [23]. Furin and TMPRSS2 are essential enzymes for S protein's cleavage at the S1/S2 and the S2′ sites, respectively [23]. Bestle et al. [23] have shown that *TMPRSS2* is necessary for SARS-CoV-2 S glycoprotein priming through *in-vitro TMPRSS2* knockdown in human epithelial cells. Similarly, they revealed that synthetic *furin* inhibitors strongly blocked the transmissibility of SARS-CoV-2 in lung epithelial cells and that more potent antiviral effects were obtained by combining *TMPRSS2* and *furin* inhibitors [23].

SARS-CoV-2 host moiety, the *ACE2* receptors, are essential for marinating human’s body homeostasis, including membrane trafficking, the renin-angiotensin (RAS), and cardiovascular systems [24], [25], [26]. *ACE2* is widely expressed in all tissues with relatively higher expression in the respiratory system, including type I and type II alveolar cells, the central nervous system, the cardiovascular system, kidneys, and the gastrointestinal tract [27]. *ACE2* acts as a key regulator of RAS, primarily by transforming Ang (angiotensin) I to Ang 1–9 and Ang II to Ang 1–7 [28]. *ACE2* activity occurs in various lung diseases, including lung injury and fibrosis, pulmonary hypertension, and ARDS [29]. More recently, the role of *ACE2* in COVID-19 pathogenesis has gained considerable attention due to its critical link with immunity, inflammation, the digestive tract, and cardiovascular diseases. SARS-CoV-2 binding affinity for *ACE2* correlates with the viral replication rate, transmissibility, and disease severity [30]. *ACE2* and viral S protein interactions are considered a promising therapeutic target for vaccine production [31].

We performed a comprehensive literature search using the PubMed/MEDLINE/ database to explore papers published until 31 January 2021. We used the following keywords: COVID-19, SARS-CoV-2, smoking, *ACE2*, *TMPRSS2*, *furin*, and treatment. Retrieved publications were selected independently for their relevance and contribution; literature search was constrained by study subjects (in-vitro, in-vivo, humans) and language (English). Furthermore, references retrieved from articles and those from recent reviews of smoking and COVID-19 were analyzed. This review also includes publications focusing on epidemiology and clinical characteristics of COVID-19, the association of smoking with COVID-19, in addition to the management and therapeutic options for COVID-19.

## 3 Smoking as a risk factor for COVID-19

Smoking is an established risk factor for several cardio-metabolic and respiratory diseases, including chronic obstructive pulmonary disease (COPD) and bronchial asthma [32]. Smoking tobacco promotes exposure to several toxic chemicals, including 1,3-butadiene, benzene, and NO_2_ [33]. It can induce inflammation of the respiratory tract, allergy, permeability of epithelial cells, mucus formation, and impair mucociliary clearance [34].

Robust evidence indicates that the risk of *Mycobacterium tuberculosis* infection is almost doubled by smoking-induced immune suppression. Likewise, the risk of influenza, *mycoplasma pneumoniae*, *legionella*, and *pneumococcal* infections is 3–5 times higher in smokers. [35]. However, literature on the role of smoking in the pathogenesis of previous coronavirus outbreaks (Middle Eastern respiratory syndrome coronavirus (MERS) and severe respiratory syndrome coronavirus (SARS)) is scarce. One study from the Republic of Korea, based only on twenty-six samples, showed that smoking increased the risk of death in MERS patients [36]. Although data is sparse for COVID-19 cases, we have summarized available data comparing disease fatality in smokers and non-smokers (Table 1).

**Table 1:** Smoking and COVID-19.

City/Country	Reported cases (%)	Smoking history		Co-morbidity (%)	Mortality (%)	Reference
		Severe cases n (%)	Mild cases n (%)			
Wuhan/China	140 (100%)	2/58 (3.4%)	0/82 (0%)	90 (64.3%)	–	[16]
Sindh/Pakistan	382 (41.5%)	Positive correlation with disease severity and death		Positive correlation	2.8%	[37]
China	1590 (100%)	111/1590 (7%)		399 (25%)	–	[38]
Wuhan/China	191 (100%)	5/54 (9%) of dead	6/137 (4%) of survivors	–	54 (28.2%)	[39]
Wuhan/China	78 (100%)	3/11 (27.3%)	2/67 (3%)	29 (37.1%)	2 (2.5%)	[40]
Chongqing/China	133 (100%)	58/65 (89.71%)	61/68 (89.23%)	133 (100%)	–	[41]
China	1099 (100%)	29/172 (16.9%)	108/913 (11.8%)	261 (23.7%)	15 (1.4%)	[42]
Wuhan/China	41 (100%)	0/13 (0%)	3/28 (11%)	13 (32%)	6 (15%)	[43]

Several COVID-19 cohort studies analyzed the impact of smoking on disease incidence, comorbidity, and mortality, the results are summarized in Table 1. The studies either reported a correlation between smoking and COVID-19 severity or divided cohorts into mild and severe cases, with percentages of smokers in each group. According to Ujjan et al. [37], there is a correlation between smoking and disease severity, with all deceased patients (2.8%) being smokers. Similarly, Zhou et al [39] studied 191 inpatients, 54 of whom died while in the hospital and 137 of whom were discharged. They found that dead patients had a greater smoking tendency than recovering ones. Although several studies found a link between disease severity and smoking [40], [42], few found no difference or a negative correlation between smoking and COVID-19 severity [41], [43].

Zhao et al. (2020) conducted a *meta*-analysis of seven studies analyzing the association of COVID-19 fatality with smoking. The authors found that smoking doubled the risk of severe COVID‐19 [44]. Similarly, a *meta*-analysis of 13 published Chinese studies found that age over 65, male gender, and smoking were risk factors for disease progression in COVID-19 patients [45]. Another *meta*-analysis conducted by Patanavanich and Glantz (2020) showed a significant association between smoking and the progression of COVID-19 [46]. Moreover, another study showed that being a smoker or former smoker was a greater risk factor for a severe COVID-19 infection (OR = 1.96, CI = 1.36–2.83) and posed a greater likelihood of a more critical condition (OR = 1.79, CI = 1.19–2.70) [32].

However, on the contrary, studies have demonstrated a lack of association between smoking, COVID-19, and the severity of COVID-19 disease. In contrast, studies carried out in China reported only 25% of the COVID-19 patients to be smokers [15], [38], [47], [48], [49], in Italy, as low as 15% of COVID-19 patients were former smokers [50].

## 4 Impact of smoking on the expression of SARC-CoV-2 key entry genes

Chronic smokers or prolonged exposure to smoke tends to display several co-morbidities, including emphysema, atherosclerosis, and immune dysregulation [51], which further enhances COVID-19 progression.

Hung et al. [52] found that exposure to smoke increased the pulmonary activity of *ACE2* and that *ACE2* knockout mice had significant pulmonary inflammation and distress in response to cigarette smoke. Smoking increased pulmonary JNK, p38, and ERK1/2 levels, indicating that *ACE2* expression caused by smoking promotes inflammation and lung injury [52]. Similarly, smoking-induced abnormal expression of the ACE2 pathway is associated with blood gas changes, lung inflammation, edema, and injury [53]. The nitrogen dioxide present in cigarette smoke enhances the binding of *ACE2* to its receptor up to 100 times by stimulating ACE enzyme activity [33]. Radzikowska et al. (2020) reported that smoking, asthma, obesity, and hypertension could elevate *ACE2* expression in the bronchial biopsy, bronchioalveolar lavage, or blood samples [54]. Similarly, Brake et al. (2020) reported that smoking upregulates ACE2 receptor expression in COVID-19 patients [55]. Further, studies have found elevated expression of *ACE2* and *TMPRSS2* in several groups of patients with chronic obstructive pulmonary disease (COPD) and idiopathic pulmonary fibrosis (IPF) [51], [56], [57], [58], [59], [60]; both expressions significantly correlated with previous cigarette exposure [56], [61], [62]; COPD is considered a major risk factor for COVID-19 [63], [44].

However, *ACE2* expression was not altered in patients with asthma or pulmonary sarcoidosis [51], [64], [65]. On the contrary, Matusiak and Schürch [66] conducted a study in asthmatic patients and found loss of *ACE2* expression in nasal epithelium and increased *TMPRSS2* expression in bronchi and central airways. Similarly, Liu et al. (2020) recorded that plasma *ACE2* level of COVID-19 patients was significantly elevated and correlated linearly with viral load and lung injury [67]. Another study by Leung et al. (2020) analyzed *ACE2* expression in the small airway epithelia of COPD patients and indicated that smoking frequency positively correlates with *ACE2* gene expression, which was higher in current smokers compared to never-smokers [68]. Since *ACE2* is considered an interferon-stimulated gene [69], corticosteroids can be attributed to one of the possible reasons for the loss of *ACE2* expression in patients with asthma. However, no significant difference was found in furin expression in both asthmatics and healthy individuals [66]. Furthermore, *in-vivo* studies also showed unaltered *ACE2* expression in a mouse model of cystic fibrosis as well as in mice exposed to several carcinogens (arsenic, ionizing radiation (IR), and 1,3-butadiene) [51], [70], [71], [72], [73].

Halwani et al. [56] used public gene expression datasets to compare the expression of *ACE2* and *TMPRSS2* in blood samples from children and adults and found no significant difference between them. There was also no difference in the expression of *ACE2* and *TMPRSS2* in COPD and diabetic patients, while there was an increase in both enzymes' expression in hypertensive patients [56]. However, in asthmatic patients, only *ACE2* expression was elevated [56]. Additionally, the expression of *ACE2* and *TMPRSS2* was lower in children's airways than smokers and patients with COPD, suggesting a plausible reason for the difference in the severity of COVID-19 disease among different groups of patients [56].

A recent study by Cai et al. [74] analyzed transcriptomic data sets regarding the association between smoking and the expression of SARS-CoV-2 receptors, *ACE2*, *TMPRSS2,* and *furin* in lung tissues. They found an upregulation of *ACE2* expression in smokers compared to non-smokers with a 25% increase in lung tissue expression [74]. Another study analyzed *ACE2* in both mice and human lung tissues and found enhanced expression of *ACE2* in smokers than non-smokers [51]. Cigarette smoke exposure increased *ACE2* expression in mice by 80 percent. Similarly, exposure to smoke in human lung epithelial cells showed increased *ACE2* expression by 30–55% relative to non-smokers [51]. Smith et al. [51] identified *ACE2* as an interferon-stimulated gene in the lung epithelium's secretory cells. They indicated that SARS-CoV-2 infections could produce positive feedback loops, further inducing *ACE2* levels and allowing viral dissemination. Moreover, Cai et al. [74] examined the effects of smoking on the pulmonary expression of *ACE2* in single bronchial epithelial cells. They found that smoking-induced morphological changes in cells of the bronchial epithelium. *ACE2* was also detected in secretory goblet cells in the airway of cigarette smokers [51], [74], whereas in non-smokers *ACE2* was only expressed in alveolar type II cells [74], [75], suggesting elevated *ACE2* expression in smokers’ lungs as a plausible derivate of smoking-induced secretory cell hyperplasia. Furthermore, single-cell sequencing data showed that *ACE2* levels were significantly associated with multiple mucin genes (*MUC1, MUC4, MUC15* and *MUC16*) and other epithelial barrier associated genes (*ALCAM*, [76] *CLDN7,* [77] and *TJP3* [78]) [51]. Also, gene ontology analysis showed that ACE2-associated transcripts correlate with genes regulating secretion, glycosylation and respond to toxic substances [79]. Besides, interferons induced significant *ACE2* expression. Although IFN-α, IFN-β, and IFN-γ treatment-induced *ACE2* expression in tracheal cells, only IFN-α and IFN-β altered *ACE2* expression in small airway cells [51], [80], [81], suggesting that *ACE2* expression can be plausibly induced by either viral infections or interferon treatment [51]. In comparison to non-smokers, the main facilitators of *ACE2* receptor in smokers are *ADAM17* [82], androgen receptor (AR) gene [83].

Another study looked at the relationship between *ACE2* expression and various smoking methods and reported that smoking but not vaping increases *ACE2* expression [84]. Furthermore, the study found that smoking nicotine and flavored e-cigarettes led to increased pro-inflammatory cytokine and inflammasome production [84]. Similarly, nicotine upregulates *ACE2* expression [85] by the activation of α7 nicotinic acetylcholine receptor (nAChR) on bronchial epithelial cells [86], [87], suggesting that smokers are at a higher risk for SARS-CoV-2 infection. Moreover, nitric oxide (NO) is also present in cigarette smoke and may impair immunity, raising the risk of SARS-CoV-2 infection [88]. Although studies have indicated NO as a risk factor for SARS-CoV-2 infection, the role of nicotine and NO in COVID-19 is controversial. In contrast, various studies indicated controversial data regarding the role of cigarette smoke regulation of *ACE2* expression. An in-vitro study analyzed cigarette smoke's effect on *ACE2* expression using human bronchial epithelial cells (H292) by an air–liquid interface system [89]. The study demonstrated that cigarette smoke exposure induced loss of *ACE2* mRNA expression and did not find any association between *ACE2* expression and release of IL-6 from H292 cells exposed to smoke; thus, suggesting that loss of *ACE2* in lung cells can be either under the direct effect of nicotine or, some other constituent in cigarette smoke [89]. Moreover, this study indicated that *ACE2* mRNA levels correlates both in smokers and non-smokers and cigarette smoking acts selectively on the bronchial epithelium by inhibiting viral infection and stimulating other genes' expression that can activate several transcriptomic pathways [89]. In addition to mRNA levels, the study analyzed protein expression of ACE2 and reported inconsistency between mRNA and protein levels of ACE2; indicating the protein expression profile is plausibly related to the fact that enhanced mRNA expression can be a consequence of compensatory mechanism for the loss of ACE2 protein expression on the cellular membrane [89]. The study also suggests a plausible pharmaceutical role of nicotine in COVID-19 treatment [89]. Few studies showed that nicotine has anti-inflammatory activity by reducing inflammation via nAChR α7 subunit on macrophages [17], [49], thus, possibly adding a therapeutic value. Similarly, NO was shown to display beneficial effects as a pulmonary vasodilator [90] and reduced the risk of severe COVID-19 disease in smokers [91].

A previous study demonstrated higher *ACE2* activity in male mouse kidneys and adipose tissues than female mice; however, the study did not report high *ACE2* expression [92]. Similarly, another study also reported higher renal *ACE2* activity in males compared to females; lower *ACE2* activity in female kidneys was found to be due to the presence of E2 in the ovarian hormone [93]. However, the study reported no sex differences in *ACE2* activity in the heart and lung [93], [94]. Moreover, research has demonstrated that sex hormones (androgens and estrogens) regulate the renin-angiotensin system [95], [96], [97], [98]; while androgens increase plasma renin activity [95], estrogens reduce plasma renin activity [96]. Based on this, Majdic (2020) proposed that sex hormones can modulate ACE2 expression in the lung. Consequently, this could be one of the underlying factors for gender disparities in COVID-19 morbidity and mortality [99]. As published, the COVID-19 death rate was found to be dependent on sex; in China [100] and Italy [101], men's death rate was significantly higher than in women. ACE2 expression was significantly lower in female primary isolated human airway smooth muscle cells than males using western blot analysis [102]. Moreover, the study demonstrated that exposure to testosterone and estrogen significantly upregulated and downregulated *ACE2* expression, respectively [102].

Moreover, sex hormonal regulation was found to regulate *ACE2* and *TMPRSS2,* resulting in differential gender susceptibility to COVID-19. While *ACE2* expression increased in females either due to skewed chromosome X inactivation or by estrogens, reduced androgen levels in women resulted in low TMPRSS2 expression increasing its protective role against COVID-19 development and progression [103]. This indicates that the role of sex hormones and chromosomes influence the discrepancy in the severity of COVID-19 infection between sexes, thus signifying the increased susceptibility to COVID-19 in men [103]. Recently, Chakladar and colleagues [83] found co-upregulation of both the SARS-CoV-2 receptors, *ACE2* and *TMPRSS2*, in smokers compared with non-smokers. The study used gene set enrichment analysis (GSEA) and found the androgen signaling pathway pertinent to *ACE2* and *TMPRSS2* expression [83]. Voinsky and Gurwitz [104] reported elevated *TMPRSS2 *expression in bronchial samples from smokers compared with non-smokers. Studies have shown that smoking enhances the androgen hormone levels [105], which increases the expression of *ACE2* and *TMPRSS2* receptors [83], [106]. Along with the stimulation of the androgen signaling pathway, upregulation of the receptors also correlated with the central regulators' overexpression (*HDAC6*, *CTNNB1*, and *SMARCA4*) of the androgen pathways [83]. Increased *TPMRSS4* expression in lung epithelial cells of smokers may be due to prolonged exposure to several compounds in tobacco smoke, including nicotine [107], acetaldehyde [108], and tar [109], resulting in oxidative stress and bronchial inflammation [110], [111], [112]. On the contrary, Cai et al. [74] did not find any correlation between smoking and *TMPRSS2* expression. Similarly, another study found that smoke-exposure enhanced *Cathepsin B*, but not *TMPRSS2* or *Cathepsin L* expression in the respiratory tract of mice and humans [51]. Moreover, analogous to lung epithelium, in smokers, *ACE2* and *TMPRSS2* expression was enhanced in the oral epithelium; thus, suggesting a high susceptibility of SARS-CoV-2 in oral epithelial cells [83].

Based on the studies reported, it is controversial whether smoking increases or reduces the risk of contracting COVID-19. A study in Atlanta performed analysis across six acute care hospitals and associated outpatient clinics in 220 hospitalized and 311 non-hospitalized COVID-19 patients and reported smoking was an independent risk factor for COVID-19 hospitalization [113]. Moreover, *meta*-analysis reported smokers to be at a higher risk of developing severe or progressive disease [114]. Unlike smoking, there is no data to support vaping prevalence in COVID-19 patients; nevertheless, it has been hypothesized that vaping could prime the lung for SARS-CoV-2 infection and correlate with worst outcomes, chiefly based on ancillary data on lung inflammatory processes described in in-vitro and in-vivo studies [115], [116]. However, epidemiological studies failed to report vaping habits in comparison to reports of smoking amongst hospitalized COVID-19 patients [117]. While, a study from the University of Stanford, based on a self-reported internet survey, revealed that use of e-cigarette increases the risk by five-fold of positive COVID-19 in comparison to never users [118], on the contrary, cross sectional self-reported surveys demonstrated lack of association between diagnosed/suspected COVID-19 among never, current, and ex-vapers [119], [120]. In addition, Farsalinos and colleagues reported that current smoking status was comparatively lower in COVID-19 patients; thus, indicating a protective role of smoking against COVID-19 [17]. Although smoking may not plausibly enhance risk for developing COVID-19, the biological and inflammatory signaling cascade that occurs during SARS-CoV-2 infection can be potentially devastating for a smoker.

## 5 Possible therapeutic targets

Drugs including chloroquine and hydroxychloroquine are currently approved for malaria and autoimmune diseases. Several countries have widely used them for COVID-19 treatment [121], [122]. However, due to their adverse side-effects, including a loss of vision, nausea, and cardiovascular complications, neither chloroquine nor hydroxychloroquine have been recommended in the clinical setting to treat COVID-19. It is shown that chloroquine and hydroxychloroquine exert their effect by accumulating in lymphocytes and macrophages, thus inhibiting pro-inflammatory cytokines secretion and activating anti-SARS-CoV-2 CD8+ T-cells [123]. Several other immunomodulators and anti-inflammatory drugs are also being tested in clinical trials [124].

On the other hand, novel therapeutic interventions are urgently needed to stop or slow down the virus's replication and spread. Many therapeutic options are currently being investigated apart from vaccine development, including drugs with distinct antiviral and immune-inflammatory properties [39], [125]. The efforts are directed to mask the COVID-19 functional receptors on human cells, *ACE2* and *TMPRSS2* [39], [125]. Several therapeutic strategies for COVID-19 have been recommended, including pre-existing medicines (chloroquine, hydroxychloroquine, remdesivir and lopinavir) for viral treatment, protein modeling, and genetic analysis using several computing techniques [126], [127], [128], [129], [130]. Camostat, the serine protease inhibitor, blocks both *TMPRSS2* and *TMPRSS4* and reduces influenza virus replication and cytokine production in primary cultures of human tracheal epithelial cells [131]. Therefore, camostat was proposed as a potential COVID‐19 therapeutic agent [132].

Reducing or quitting smoking can slow down the damage of the respiratory epithelial architecture [133], reduce secretory cell hyperplasia [134], and decrease *ACE2* secretion by 40% [51]. Soluble ACE2 is considered a plausible treatment option in cases where *ACE2* is over-expressed in the airway's epithelium in smokers [135]. Monteil et al. [136] showed that human recombinant soluble ACE-2 (hrsACE2) reduced SARS-CoV-2 viral loads in infected Vero-E6 cells, kidneys, and vascular organoids. In Europe, hrsACE2 is under Phase 2 clinical trial (ClinicalTrials.gov NCTO4335136) as a therapeutic agent for COVID-19 [137]. However, the efficacy of such therapy in smokers and COPD patients who have elevated levels of *ACE2* needs to be determined. Moreover, use of therapeutics and dietary supplements to normalize NO levels in smokers should be considered to minimize the incidence of COVID-19 disease and its complications [88].

Investigations using *in-vitro* models and anti-retroviral drugs (lopinavir and ritonavir) are effective against human immunodeficiency virus 1 (HIV-1) infection, SARS-CoV, MERS-CoV, and SARS-CoV-2 viruses [138], [139]. However, research has shown that neither lopinavir nor ritonavir improves symptoms compared to standard care in severe COVID-19 patients [140], [141]. However, a combination of lopinavir-ritonavir with oseltamivir showed complete recovery after COVID-19 pneumonia [142], [143]. On the other hand, remdesivir has been shown to effectively inhibit SARS-CoV-2 in *in-vitro* studies using Vero E6 cells [126], [144].

## 6 Summary and outlook

Although smoking can enhance the expression of key entry genes of SARS-CoV-2 used for viral activation, the underlying mechanisms of tobacco-related upregulation of these receptors and the degree to which smoking affects infection susceptibility and clinical manifestations remain unknown. Future mechanistic studies are warranted to address these issues.

## Funding source

This research did not receive any specific grant from funding agencies in the public, commercial, or not-for-profit sectors.

## Ethical Approval

None.

## Declaration of Competing Interest

The authors declare that they have no known competing financial interests or personal relationships that could have appeared to influence the work reported in this paper.
